# Analysis of organoid and immune cell co-cultures by machine learning-empowered image cytometry

**DOI:** 10.3389/fmed.2023.1274482

**Published:** 2024-01-17

**Authors:** Philipp Stüve, Benedikt Nerb, Selina Harrer, Marina Wuttke, Markus Feuerer, Henrik Junger, Elke Eggenhofer, Bianca Lungu, Simina Laslau, Uwe Ritter

**Affiliations:** ^1^Division of Immunology, LIT – Leibniz Institute for Immunotherapy, Regensburg, Germany; ^2^Chair for Immunology, University of Regensburg, Regensburg, Germany; ^3^Department of Surgery, University Hospital Regensburg, Regensburg, Germany; ^4^TissueGnostics SRL, Iași, Romania

**Keywords:** organoid, lymphocytes, co-culture, imaging, Matrigel^®^

## Abstract

Organoids are three-dimensional (3D) structures that can be derived from stem cells or adult tissue progenitor cells and exhibit an extraordinary ability to autonomously organize and resemble the cellular composition and architectural integrity of specific tissue segments. This feature makes them a useful tool for analyzing therapeutical relevant aspects, including organ development, wound healing, immune disorders and drug discovery. Most organoid models do not contain cells that mimic the neighboring tissue’s microenvironment, which could potentially hinder deeper mechanistic studies. However, to use organoid models in mechanistic studies, which would enable us to better understand pathophysiological processes, it is necessary to emulate the *in situ* microenvironment. This can be accomplished by incorporating selected cells of interest from neighboring tissues into the organoid culture. Nevertheless, the detection and quantification of organoids in such co-cultures remains a major technical challenge. These imaging analysis approaches would require an accurate separation of organoids from the other cell types in the co-culture. To efficiently detect and analyze 3D organoids in co-cultures, we developed a high-throughput imaging analysis platform. This method integrates automated imaging techniques and advanced image processing tools such as grayscale conversion, contrast enhancement, membrane detection and structure separation. Based on machine learning algorithms, we were able to identify and classify 3D organoids within dense co-cultures of immune cells. This procedure allows a high-throughput analysis of organoid-associated parameters such as quantity, size, and shape. Therefore, the technology has significant potential to advance contextualized research using organoid co-cultures and their potential applications in translational medicine.

## 1 Introduction

Cell-based assays are invaluable tools in clinical research for studying the interactions between immune, stromal, and parenchymal cells *in vitro*. However, most of these *in vitro* models cannot entirely mimic complex *in vivo* processes since 2D cell-monolayer models do not contain a tissue-specific microenvironment (1). During the last decades, stem cell- or adult tissue progenitor cell-derived organoids have emerged as suitable *in vitro* applications in translational research of human chronic diseases (2, 3). Organoids are self-organized, 3D multicellular tissue cultures, serving as artificial model systems of organs (2, 4–7). They can be generated *in vitro* from almost every murine and human tissue, such as the liver, intestine, and brain (8). Since organoids also resemble many features of native human organs, such as functionality and structure, they are also referred to as “mini-organs” (2).

A requirement for conducting mechanistic studies with organoids is to develop experimental systems that accurately replicate the functional and structural complexity *in vivo*. Various scientific techniques, such as molecular analysis, gene editing, and imaging, can be used to characterize functional organoid models (9). Based on these experimental protocols, organoid technology offers a wide range of immunological applications, varying from basic research, including the analysis of tissue biology, tumor immunology, and host-pathogen interactions, to screening of drugs in regenerative medicine (3).

However, classical organoid cultures lack cells from the adjacent tissue microenvironment, such as immune, parenchymal, and stromal cells, thus potentially limiting mechanistic studies. Therefore, co-culturing organoids with cells from their adjacent tissue is necessary to gain a higher level of physiologically contextualized organoid research (3).

Several experimental *in vitro* systems are currently available to investigate the involvement of immune cells in stem cell development and organoid formation (reviewed in (3)). In the context of tumor research, tumor organoids can be used either for the identification of tumor-reactive T cells (10) or for testing organoid-cytolysis induced by chimeric antigen receptor (CAR)-engineered lymphocytes in combination with immune checkpoint inhibitors (11–13). Other co-culture systems of organoids, which simulate the microenvironment of the intestinal lamina propria, are also used to investigate the crosstalk between intestinal immune cells and epithelial stem cells in the context of tissue development or inflammation (14–18). Most recently, a relevant interaction between T cells and intestinal stem cell development has also been proposed (15, 17, 18).

Organoid co-cultures may also be a promising tool for basic and translational research, as potential communication between organoids and other cell subsets can be investigated. This setup would allow the characterization of immune- or parenchymal-derived factors that are expected to modulate organoid development. To this end, high-throughput brightfield imaging of the entire culture wells can be performed, generating time-lapse and end-point analyses (19). However, the quantification of experimental parameters, such as organoid number, size, and shape, still remains challenging due to the following reasons: Firstly, 3D stereoscopic organoid cultures are embedded in Matrigel® or other appropriate extracellular matrix gels, such as Hydrogel® or Geltrex® (20). Thus, organoids grow at different focus levels and may appear out of focus under fixed focus conditions during microscopy (21). Secondly, due to their heterogeneous differentiation status, the organoids have different shapes and dimensions (21). Thirdly, dense cell clusters of proliferating immune cells are similar to organoids and have the potential to generate interfering imaging signatures, leading to false positive results.

Most of the previously published image-processing algorithms were developed for the analysis of organoid cell cultures in the absence of additional cell subsets (compare Table 1). To date, no high-throughput image analysis workflow is published that are capable of identifying and quantifying organoids within co-cultures. Thus, we developed an organoid detection application (Organoid App), which provides a reliable and effective tool for the high-throughput identification, validation, and quantification of organoids in co-cultures with immune cells. In order to realize this methodological project, we used extrahepatic cholangiocyte organoid (ECO) cultures (6, 26), which served as a model system for studying homeostasis and regeneration as they contain both stem cells and differentiated epithelial cells (27). These ECOs were co-cultured with polarized human effector T cells. For the development of our Organoid App, we used the commercially available StrataQuest image cytometry platform.

**Table 1:** Organoid detection pipelines.

Name	Organoid model	Imaging pipeline	Organoids analysis	Co-culture	Ref.
OrganoSeg	Colorectal cancer Pancreas	Grayscale images (brightfield, phase-contrast, differential-interference contrast) One Focus level	Size, distributions and morphology	No	(22)
OrgaQuant	Intestinal epithelium	Grayscale images (brightfield) One Focus level	Diameter in pixel	No	(23)
OrganoidTracker	Small intestinal epithelium	Immune fluorescent labeled H2B-mCherry One Focus level	Tracking	No	(24)
DNN	Alveolar	Grayscale images (brightfield) Merged z-stacks	Tracking number	No	(21)
OrganoID	Pancreatic cancer	Grayscale images (brightfield) One Focus level	Area tracking	No	(25)
D-CryptO	Colon culture	Grayscale images (brightfield) Merged z-stacks	Size diameter number	No	(1)

In summary, our Organoid App enables the exploration of complex questions concerning the influence of human immune cell subsets and other compounds on organoid growth and development. This advancement offers great potential for addressing challenging applications in the field of translational medicine.

## 2 Materials and equipment

Detailed information about material and equipment is summarized in Table 2. Materials including hardware components and *in vitro* culturing of organoids are described below.

**Table 2:** Key resources.

Biological samples: recombinant proteins		
Reagent or resource	Source	Identifier
human R-Spondin 1	Peprotech	Cat. No.: 120-38
human EGF	Peprotech	Cat. No.: AF-1000
human HFG	Peprotech	Cat. No.: 100-39
human FGF-10	Peprotech	Cat. No.: 100-26
human EGF-10	Peprotech	Cat. No.: AF-1000
IL-2	Novartis	Proleukin® S
Software and Algorithms		
OrganoSeg	Borten et al.,	https://github.com/JanesLab/OrganoSeg
StrataQuest v7.1.1.138	TissueGnostics	https://tissuegnostics.com/products/contextual-image-analysis/strataquest
Incucyte® Organoid Analysis ModuleIncucyte	Sartorius	https://www.sartorius.com/en/products/live-cell-imaging-analysis/live-cell-analysis-software/incucyte-organoid-analysis-software
TissueFAXS-plates software v7.1.6245.120	TissueGnostics	https://tissuegnostics.com/products/scanning-and-viewing-software/tissuefaxs-imaging-software
GraphPadPrism 9.5.1 for macOS	GraphPad Software, LLC.	https://www.graphpad.com
Plastic material		
48-well plate	Corning/Merck KgaA	Cat. No.: CLS3548
CellStrainer	Miltenyi	Cat. No.: 130-098-462
Critical cell culture components		
Matrigel®	Corning	Cat. No.: 356230
HEPES	PAN-Biotech	Cat. No.: P05-01100
L-Glutamin	PAN-Biotech	Cat. No.: P04-80100
Antibiotic-antimycotic	Gibco	Cat. No.: 15240062
N_2_ serum-free supplement	Gibco	Cat. No.: 17502-048
B-27 serum-free supplement	Gibco	Cat. No.: 12587-010
N-Acetyl L-Cystein	Sigma/Merck KgaA	Cat. No.: A9165
Gastrin I	Sigma/Merck KgaA	Cat. No.: G9145
Nicotinamin	Sigma/Merck KgaA	Cat. No.: N0636
A83-01	BioGems/ Hölzel Diagnostika GmbH	Cat. No.: 9094360
Forskolin	R&D/Bio-Techne	Cat. No.: 1099
Y-27632	BioGems/ Hölzel Diagnostika GmbH	Cat. No.: 1293823
DPBS	Gibco	Cat. No.: 14190-094
TexMACS™ Medium	Miltenyi Biotec	Cat. No.: 130-097-196
T Cell TransAct™	Miltenyi Biotec	Cat. No.: 130-111-160
ADV/DMEM-F12	Gibco	Cat. No.: 12634010
EBSS	Gibco/Thermo Fisher Scientific Inc.,	Cat. No.: 24010043
Collagenase	SIGMA/Merck KgaA	Cat. No.: C9891
Cell separation reagents		
Pancoll®	PAN-Biotech	Cat. No.: P04-601000
biotinylated anti-human CD8 (clone HIT8a)	BioLegend	Cat. No.: 300904
anti-biotin ultrapure microbeads	Miltenyi Biotec	Cat. No.: 130-105-637
Imaging hardware		
TissueFAXSiPlus	TissueGnostics	https://tissuegnostics.com/products/fluorescence-brightfield-cytometer/tissuefaxs-i-plus
Incucyte® SX5 Live-Cell Analysis System	Sartorius	https://www.sartorius.com/en/products/live-cell-imaging-analysis/live-cell-analysis-instruments/sx5-live-cell-analysis-instrument?&utm_source=google&utm_medium=cpc&utm_campaign=incucyte&utm_term=brand&utm_content=search&gad=1&gclid=CjwKCAjw-7OlBhB8EiwAnoOEk3ycK0ZXXZanqBUwI_8aUyKfrOQNgP3NbvxddlEF7IIGWCaaQMbVhRoC0TsQAvD_BwE

### 2.1 Hardware and software components

**Figure 1:**
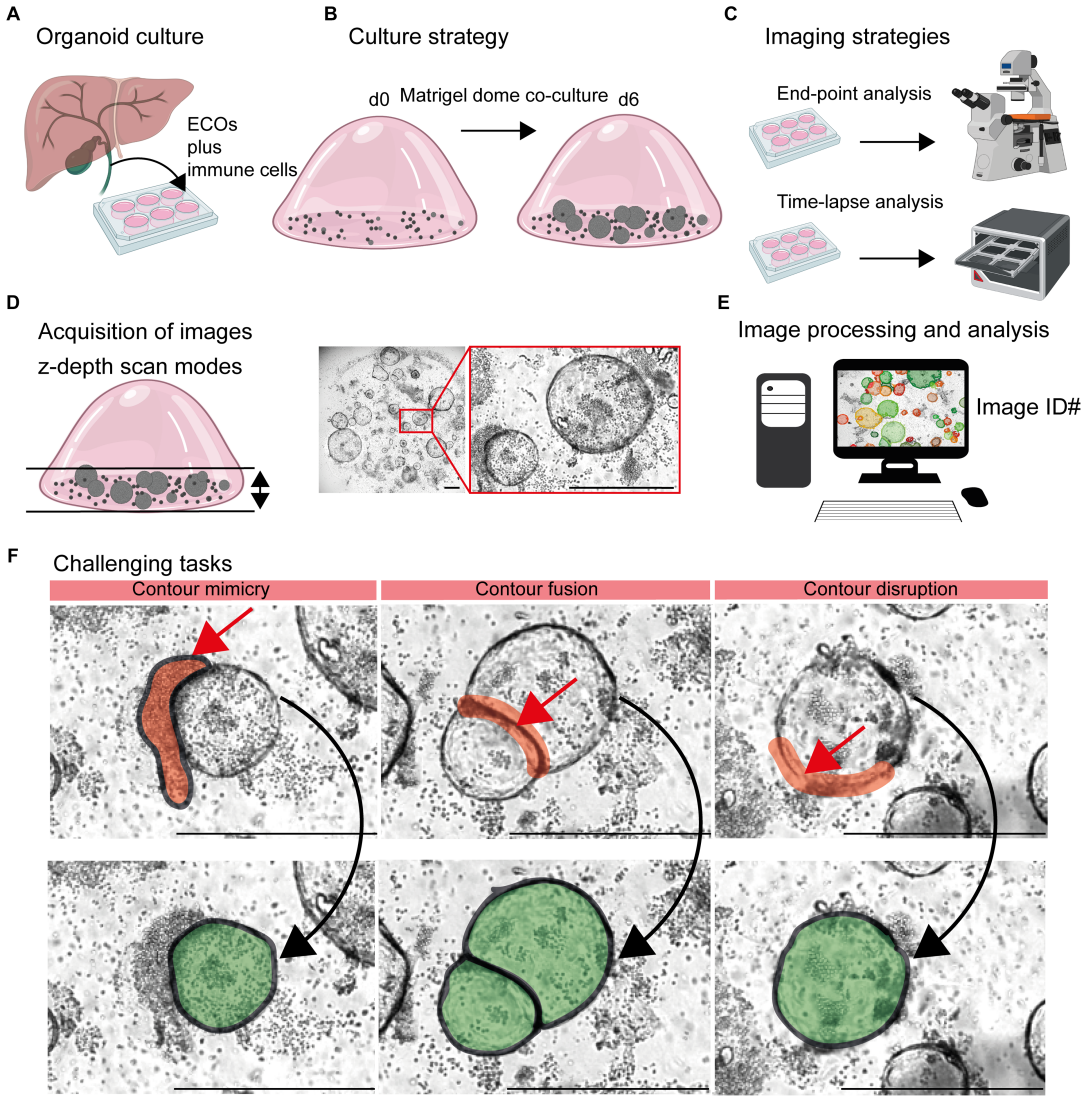
Detection of organoids in co-cultures of organoids and immune cells. **(A)** Organoid culture: Extrahepatic cholangiocyte organoids (ECOs) were generated from gallbladder tissue. **(B)** Culture strategy: ECOs and immune cells embedded in Matrigel® were co-cultured for a period of 6 days. Details are given in Section 2.2 and 2.3. **(C)** Imaging strategies: Culture plates are incubated within cell incubators for end-point analysis or the Incucyte® system, allowing an incubation and imaging for a period of 6 days. **(D)** Acquisition of images: Capturing red, green and blue (RGB) images with z-depth enables the generation of images with high plasticity. **(E)** Image processing and analysis: Import of images and image identification information (ID: well number and time point) in suitable devices for subsequent detection and quantification of organoids by the StrataQuest-supported Organoid App. **(F)** Challenging tasks: The main challenging tasks of organoid detection are depicted. Upper row: Red arrows indicate contour mimicry (left), contour fusing (middle) or contour disruption (right). Red areas highlight the regions that might interfere with a precise detection of organoids. Lower row: The aimed precision of organoid detection is highlighted in green. The bars represent 500 μm.

End-point analysis of organoid co-cultures (Figures 1A,B) were conducted using the automated TissueFAXSiPLUS (TissueGnostics, Vienna, Austria; Objective: EC Plan Neofluar 5x/0,25 M27, Zeiss, Oberkochen, Germany) system, including scanning with the appropriate TissueFAXS-plates software module (TissueGnostics). The time-lapse image acquisition was performed with the Incucyte® SX5 Live-Cell Analysis System (Sartorius, Goettingen, Germany) (Figure 1C). The Incucyte® Organoid Analysis Module (Cat. No. 9600-0034) was operated with the following settings: segmentation radius of 200 μm, segmentation sensitivity of 50, segmentation edge split with edge sensitivity of 70, cleanup with hole fill of 500,000 μm^2^ and adjusted pixel size of −4. Additionally, filters for organoid quantification were set for a minimal area of 19,000 μm^2^ and maximal eccentricity of 0.8. Image analysis by the OrganoSeg (22), software was conducted according the instructions given by the developer^1^ (28). As mentioned in the OrganoSeg manual^2^ (28), the following settings were used for optimal organoid detection. Given the fact that the ideal parameters of “Intensity threshold” and “Window size” vary from image to image, they had to be adapted for each individual image. Images with different levels of complexity were analyzed according the following parameters: Level #1 – Segmentation, “Out-of-focus correction” (default: ON), “Differential interference contrast (DIC) correction” (default: OFF); “Intensity threshold” 0.1172; “Window size” 90; “Size threshold” 699. Level #2: – Segmentation, “Out-of-focus correction” (default: ON), “DIC correction” (default: OFF); “Intensity threshold” 0.82987; “Window size” 20; “Size threshold” 513. Level #3 – Segmentation, “Out-of-focus correction” (default: ON), “DIC correction” (default: OFF); “Intensity threshold” 1; “Window size” 100; “Size threshold” 699. After automatic segmentation, no organoids were manually removed or combined using “Spheroid editing toolbar.” Analysis desktop configuration: device name DESKTOP-R2MMV91, Processor Intel(R) Xeon(R) W-2133 CPU @ 3.60 GHz 3.60 GHz, Installed RAM 24.0 GB (23.7 GB usable), System type 64-bit operating system, x64-based processor, Edition Windows 10 Pro for Workstations.

### 2.2 Generation of extrahepatic cholangiocyte organoids (ECOs)

For organoid generation (Regensburg University, ethical committee, reference 16-101-5-101), gallbladder tissues (2 cm^2^ or less) were washed twice with cold Earle’s Balanced Salt Solution (EBSS; Cat. No.: 24010043, Gibco/Thermo Fisher Scientific Inc., Schwerte, Germany), cut into small pieces, and digested in 4 mL digestion solution: 25 mg/mL Collagenase from clostridium histolyticum (Cat. No.: C9891-100 mg SIGMA/Merck KGaA, Darmstadt, Germany) in EBSS for 20 min at 37°C with soft shaking and filtered through a 70 μM Nylon CellStrainer (Cat. No.: 130-098-462 Miltenyi, Bergisch Gladbach, Germany). Dissociated cells were centrifuged at 1500 rpm (470 g) for 5 min at 4°C and washed twice with Base-medium (antibiotic-antimycotic 100x, Cat. No.: 15240-062, Gibco/Thermo Fisher Scientific Inc., New York, USA, 100 U/mL penicillin, 100 μg/mL streptomycin, AmphotericinB 1 μg/mL, L-Glutamine 2 mM Cat. No.: G7513-100 mL SIGMA/Merck KGaA, Darmstadt, Germany, HEPES 50 mM Cat. No.: H0887-100 mL, SIGMA/Merck and Advanced DMEM F12 Cat. No.: 12634028, Gibco/Thermo Fisher Scientific Inc). Organoid cultures were established according to previously published methods (6, 26). In brief, cell pellets were resuspended in organoid culture medium mixed with Matrigel® (Cat. No.: 356230 Corning, Corning, New York, United States of America) in a 50/50 ratio. Matrigel® was allowed to solidify for 15 min at 37°C before adding organoid culture medium. Organoid culture medium was based on ADV/DMEM-F12 (Cat. No.: 12634010, Gibco) supplemented with 1 M Hepes (Cat. No.: P05-01100, PAN-Biotech, Aidenbach, Germany), 100 mM L-Glutamin (Cat. No.: P04-80100, PAN-Biotech), 3.6% Anti-Anti (Cat. No.: 15240062, Gibco, Fisher Scientific GmbH, Schwerte, Germany), 1% N_2_ serum-free supplement (Cat. No.: 17502-048, Gibco) 1% B27 serum-free supplement (Cat. No.: 12587-010, Gibco), 1 mM N-Acetyl L-Cystein (Cat. No.: A9165, Sigma/Merck KGaA, Darmstadt, Germany), 10 nM Gastrin I (Cat. No.: G9145, Sigma/Merck KGaA) and the following growth factors: 1 μg/mL of recombinant human R-spondin 1 (Cat. No.: 120-38, Peprotech, Hamburg, Germany), 10 mM Nicotinamin (Cat. No.: N0636, Sigma/Merck KGaA), 5 μM A83-01 (Cat. No.: 9094360, BioGems/Hölzel Diagnostika GmbH, Hohenzollernring, Germany), 10 μM Forskolin (Cat. No.: 1099, R&D/Bio-Techne, Wiesbaden, Germany), 50 ng/mL human epidermal growth factor (EGF, Cat. No.: AF-1000 Peprotech), 50 ng/mL human hepatocyte growth factor (HFG, Cat. No.: 100-39, Peprotech) and 100 ng/mL human fibroblast growth factor-10 (FGF-10, Cat. No.: 100-26, Peprotech). For the first 72 h after thawing, 10 μM of Y-27632 (Cat. No.: BioGems/Hölzel Diagnostika GmbH) was added to the media and only 25 ng/mL of HGF was used. Medium was changed every 3–4 days. Organoids were split every week by mechanical dissociation into small fragments and transferred to fresh Matrigel®.

### 2.3 Organoid co-culture with immune cells

For the isolation of CD8^+^ T cells from human blood, leukocyte reduction chambers (provided by Transfusion Medicine, University Hospital Regensburg; ethical committee, reference number 13-0240-101 and 19-1414-101) were used. Leukocytes were initially diluted three times in DPBS (Cat. No.: 14190-94, Gibco), and the resulting blood and PBS mixture was split in two fractions and underlaid with an equal amount of Pancoll® (Cat. No.: P04-601000, PAN-Biotech, PAN-Biotech GmbH, Aidenbach, Germany). Samples were centrifuged at 1,000xg for 20 min at RT, with acceleration set to four and brake to zero. The PBMC layer was isolated and washed twice by centrifugation steps. CD8^+^ T cells were isolated by column-based magnetic separation using biotinylated anti-human CD8^+^ (clone HIT8a, Cat. No.: 300904 Biolegend, Koblenz, Germany) and anti-biotin ultrapure microbeads (Cat. No.: 130-105-637, Miltenyi Biotec, Bergisch Gladbach, Germany), followed by fluorescence-activated cell sorting of viable CD8^+^ T cells.

100,000 cells/well were seeded in TexMACS™ medium (Cat. No.: 130-097-196, Miltenyi Biotec) supplemented with 1% Penicillin/Streptomycin and activated with T Cell TransAct™ (1:100) (Cat. No.: 130-128-785, Miltenyi Biotec) in the presence of a cytokine mix for effector T-cell differentiation. Due to ongoing confidential work on this topic, further details about the T-cell phenotype cannot be provided. The absence of this confidential information is not relevant to the presentation of the developed Organoid App and will be discussed in follow up studies in detail.

After 4 days, CD8^+^ effector T cells were harvested and rested in TexMACS™ medium with 100 U/mL IL-2 (Proleukin® S Novartis, Nürnberg, Germany) for three days. Subsequently, 200,000 T cells were stimulated with T Cell TransAct™ (Miltenyi Biotec) o/n before harvesting the cells. Organoids were harvested and washed with PBS to remove Matrigel®. For co-culture experiments, organoids and effector T cells were mixed in a ratio of 20 organoids/10,000 effector T cell in organoid culture medium without growth factors. The mixes were pelleted and resuspended in a 50/50 mixture of Matrigel® and organoid culture medium supplemented with growth factors as described above. Then, 25 μL of the mixture was seeded in the center of the wells of a 48-well plate (Cat. No.: CLS3548, Corning/Merck KGaA) and incubated at 37°C for 15 min to allow matrix solidification and dome formation. Finally, 300 μL of organoid culture medium supplemented with growth factors and 100 U/mL IL-2 was added and co-cultured by 37°C and 5% CO_2_ in the Incucyte® SX5 Live-Cell Analysis Instrument (EssenBioscience/Sartorius, Göttingen, Germany). Scans of the individual wells were scheduled every 8 h over a period of 6 days. To assess the influence of the growth factor EGF on organoid growth, organoid co-cultures were cultured with or without 50 ng/mL human EGF.

### 2.4 Statistical analysis of data

Raw data (*.xls or *.xlsx) were imported into GraphPadPrism 9 macOS for subsequent graphical presentation and statistical analysis. Multiple *t*-tests (Multiple Mann–Whitney tests; unpaired; nonparametric), one way ANOVA tests (Kuruskal-Wallis test, Dune’s multiple comparison) and Šídák’s multiple comparisons test were used for statistical analysis.

### 2.5 Calculation of precision and recall

For the evaluation of the potential of software’s to detect organoids, we verified the software-based results manually be counting true positive (TP), false positive (FP) and false negative (FN) signals. The parameters precision (positive predictive value) and recall (sensitivity) have been calculated according the formula: Precision = TP/(TP + FP); Recall = TP/(TP + TN) (29). Consequently, the following parameter associated questions could be addressed. Precision: What proportion of the positive identifications were actually correct? Recall: What proportion of true positives was correctly identified?

## 3 Methods

### 3.1 Probing technical limitations of automated detection and quantification of organoids in co-cultures

The influence of immune cells on the growth of organoids is gaining more and more acceptance in the field (3, 8, 11–18, 30, 31). In this study, we chose the ECO organoid model in combination with lymphocytes embedded in Matrigel® domes to generate time-lapse and end-point images for subsequent analysis (Figures 1A–E).

Based on the 3D structure of Matrigel® domes, a combination of multiple images taken at different focus distances was performed [TissueFAXSiPLUS: z-stacks, *n* = 4, range 310 μm; Incucyte®: brightfield organoid scan mode, object driven focus, z-depth < 2.9 mm (32)]. This allows for a high-contrast visualization of both organoids and lymphocytes embedded within different layers of the Matrigel® dome (Figure 1D; Supplementary Figure S1).

Based on these initial data, an accurate detection of organoids within heterogeneous and dense *in vitro* cultures remains challenging due to three main aspects (Figures 1D, F). Firstly, clusters of immune cells that are located close to organoid structures can mimic organoid morphology based on their cellular density. Therefore, the detection algorithms could recognize false positive structures based on contour mimicry. Secondly, when organoids are in close proximity to each other, the algorithm may recognize them as a ‘single organoid structure’ due to contour fusion, which underestimates the actual organoid number. Thirdly, the integrity of organoid contours can be disrupted due to inadequate imaging quality or the presence of overlapping immune cells.

To address these challenges, high-throughput software pipelines that can accurately detect organoid structures in complex samples would be a valuable asset in studying the immunological aspects of organoid growth. Several software tools have been released to identify and characterize organoids cultured in the absence of other cellular components (Table 1). Based on our knowledge, no imaging workflows have been developed to quantify organoid-immune cell co-culture systems in a high-throughput manner. Thus, we questioned whether existing codes or commercial software tools can distinguish between organoid structures and densely clustered immune cells.

**Figure 2:**
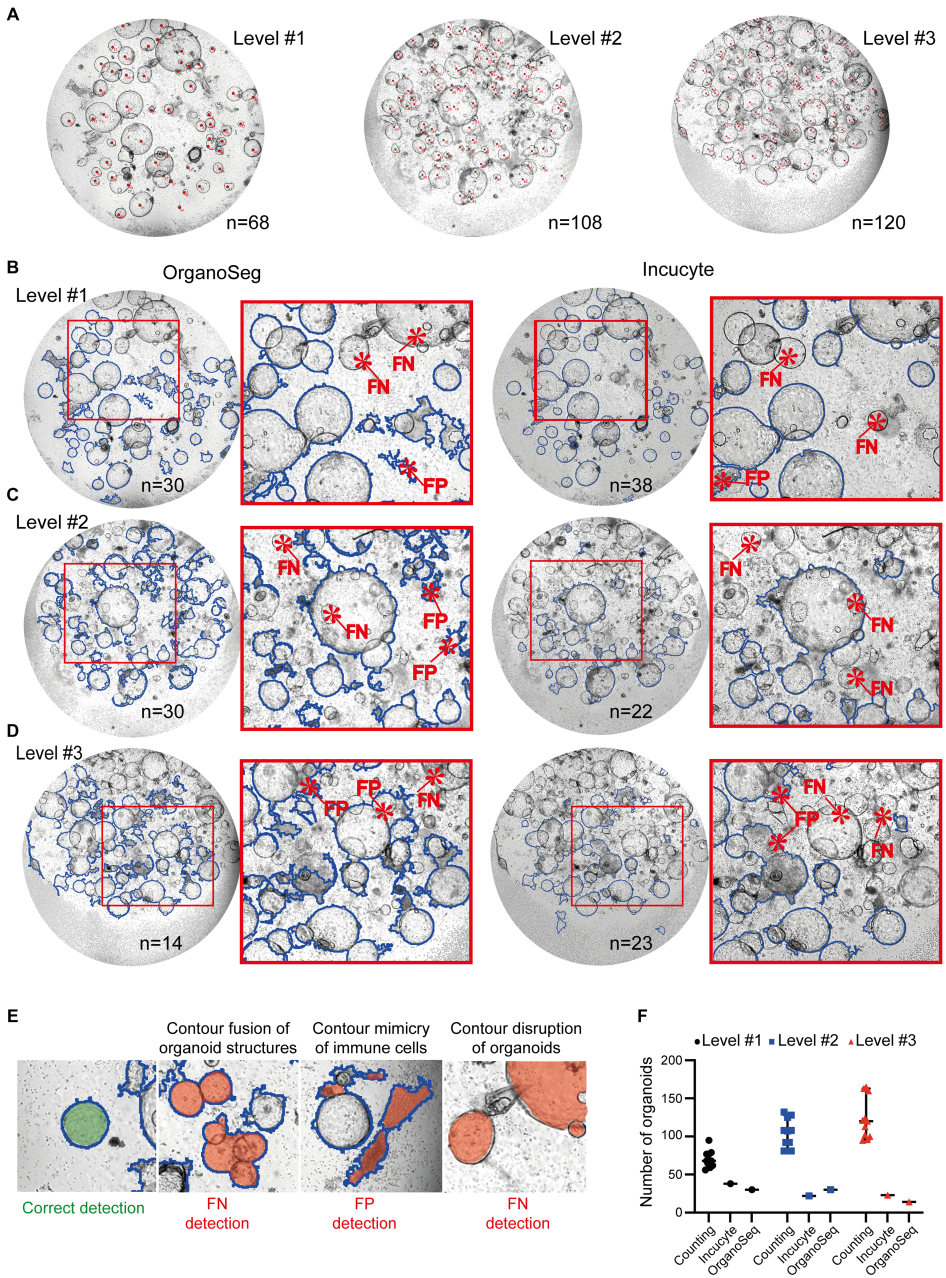
Quantification of organoids within immune cell co-cultures. Images of immune cell/organoid co-cultures representing different degrees of complexity (levels #1-#3) have been analyzed. **(A)** Organoids from level #1 – #3 were counted manually by four different scientists using the ImageJ counting function or written notes on the original pictures. Each image was counted 11 times. Representative images with organoids marked by red dots are shown. The depicted number represents the median number of organoids within the field of view. **(B–D)** Automated analysis of different degrees of complexity (levels #1-#3). Left: Images were analyzed using OrganoSeq. Right: Images were analyzed using the manufacturers’ Incucyte^®^ Organoid Analysis Module. Blue outlines visualize organoids which are detected by the respective software. Detailed software settings are described in Section 2.1. Red highlight false negative (FN) or false positive (FP) detections of organoids. **(E)** Representative examples of correct organoid detections (blue outline; filled with green). The filled red area indicates “organoid structures” that are detected FN or FP. **(F)** Comparison of organoid numbers, analyzed by the different software tools (counting: median plus 95% confidence interval is shown; Incucyte^®^ and OrganoSeg analysis: each dot represents an analysis).

Scientists can quantify organoids using different tools, such as ImageJ or handwritten notes. These results are quite accurate, but can also show variations, depending on who counted the organoids (Figures 2A,F). However, it is exceedingly challenging and time consuming to precisely determine the organoid’s area and density utilizing manual techniques. Furthermore, this manual approach is unsuitable for high-throughput analyses. We used automated approaches, such as customized Incucyte® software packages and the OrganoSeg (22) detection algorithm, to identify and quantify organoids within co-cultures. Images of increasing complexity (level #1 – #3) have been included in this study. Our findings showed that the detection of organoids by both existing software tools is not optimized for heterogeneous co-cultures. All analyzed images, ranging from intermediate (level #1; Figure 2B), to high (level #2; Figure 2C), to very high complexity (level #3; Figure 2D) showed false negative (FN) and/or false positive (FP) organoid structures. The most prominent FP and FN detection problems are given in Figure 2E. In comparison to manual counting by ImageJ (*n* = 68 in level #1, *n* = 108 in level#2, *n* = 120 in level #3), the Incucyte® and OrganoSeg software tools only highlighted a few (*n* < 39) organoid structures (Figure 2F). The calculated values for precision and recall, which are basic concepts for evaluating the performance of detection algorithms, show very low levels (Incucyte®: level #1 precision = 0.43/recall = 0.43, level #2 precision = 0.00/recall = 0.00, level #3 precision = 0.10/recall = 0.042; Organoseg: level #1 precision = 0.33/recall = 0.37, level #2 precision = 0.06/recall = 0.08, level #3 precision = 0.00/recall = 0.00; data not shown). This indicates very strong limitations regarding the detection of organoids. Despite the fact that the automated tools failed to accurately identify organoids, we plotted the number of “organoid structures” that were identified by the software (Figure 2F). In line with the unacceptable levels of recall and precision, those organoid numbers do not reflect the real situation (Figure 2F). Thus, we can conclude that the tested software tools or algorithms are limited in their ability to correctly recognize organoids under co-culture conditions (Figure 2). Therefore, our objective was to develop a high-throughput pipeline for the identification and quantification of organoid structures in lymphocyte co-cultures.

### 3.2 Image acquisition and data import for subsequent organoid detection by the StrataQuest-supported Organoid App

**Figure 3:**
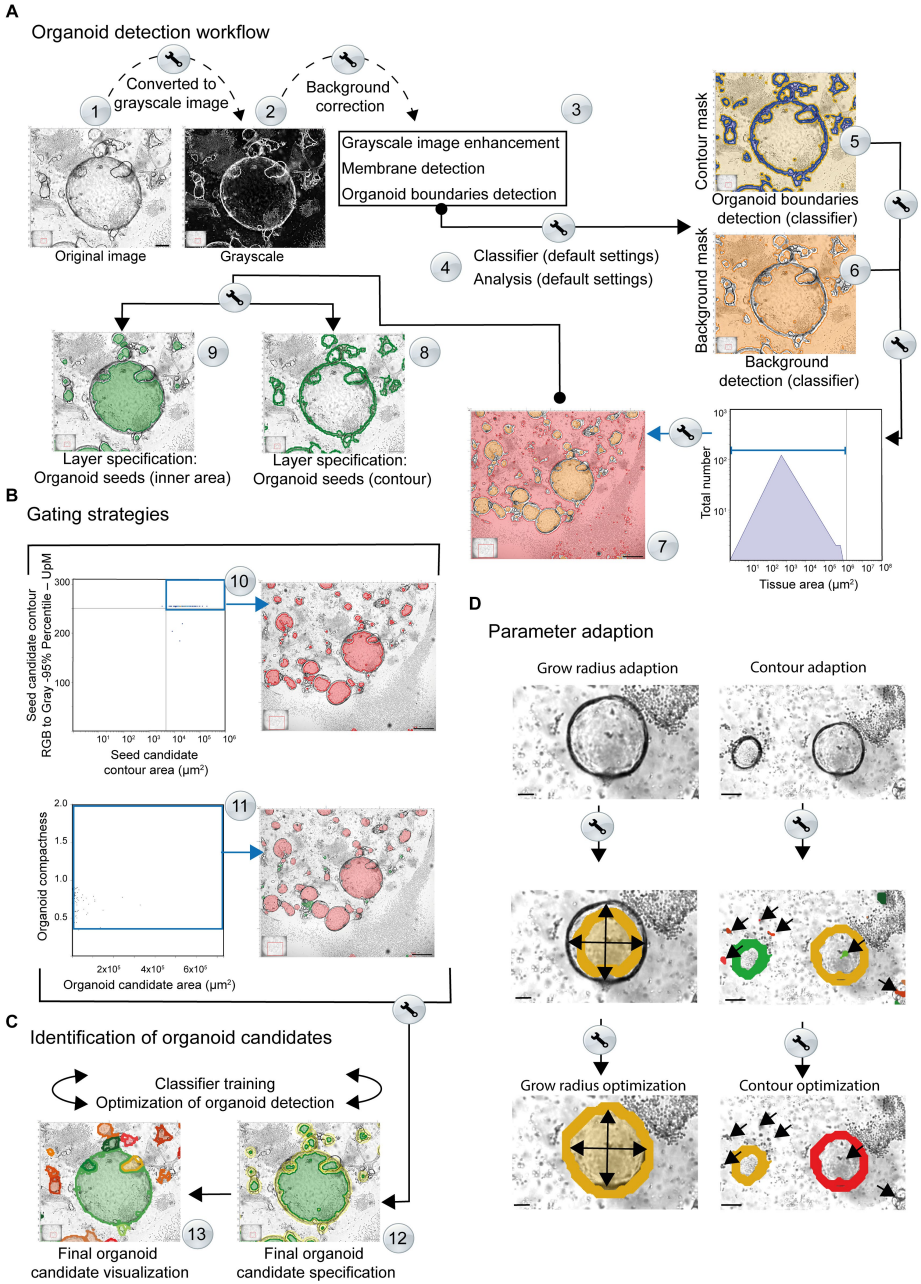
Simplified overview of the organoid detection workflow using the StrataQuest-powered Organoid App. The principal steps are depicted. Details are given in the main text. **(A)** After converting the original images to grayscale, a median filter is applied to create a background layer, which is then subtracted from the grayscale image (Step #1). This step helps to reduce interfering signals and eliminates non-uniform and high background signals (Step #2). An additional membrane detection step is integrated, for accurate discrimination of the organoid boundary (Step #3). The enhanced grayscale image is subjected to the machine learning procedure (classifier; Step #4) to identify the contours (contour mask, highlighted in blue) as well as the tissue or background (background mask; highlighted in orange). This analysis provides a boundary specification for the organoids (contours mask: blue area, background mask: orange area). By combining the input data from steps #5 and #6, a final organoid specification can be performed. Organoid boundaries (blue areas) and background (orange areas) are separated for individual images (Steps #5 and #6) and are further processed. Organoid candidates are generated (highlighted in orange, Step #7). A two-layer specification of the contour of the organoid cores (Step #8, highlighted by green lines) and the inner region of the organoids (Step #9, highlighted by filled green areas) is used for further recognition of the organoids. **(B)** Gating strategies. Representative gating and back gating tools for quality controls are depicted. Upper row: The histogram shows the range of structures that are processed by the software for subsequent organoid detection. The gate (blue square) captures signals that serve as basis for further evaluation. The back-gating function can be used to highlight (orange) all structures representing the defined gate in the original image (Step #10). Lower row: The organoid compactness signature is used to define the final detection parameters (Step #11). The gate (blue square) captures all signals that serve as final basis for quantification of organoid area, number and shape. The bar represents 500 μm. **(C)** Identification of organoid candidates. Representative specifications are depicted (Step #12). The organoid contours (yellow) and areas (green) are highlighted. Individual organoids are visualized by the software by randomly selected colors (Step #13). **(D)** Parameter adaption. Right side: the growing masks of organoid seeds and the corresponding limits of the growth radii can be adjusted until the exact limit of the organoid candidates is reached. Upper image: original. Middle image: the organoid detection (orange area) based on default growing parameters is shown. The arrows highlight the growing mask. An adaption of the organoid seeds’ grow radius (value = 280.00) and the organoid grow radius (value = 90.00) revealed an optimal detection of the organoid area (filled inner orange area). The bars represent 50 μm. Right side: the contour areas of organoid candidates can be adapted. Upper image: original. Middle image: the organoid detection (orange and blue contour line) based on default settings of contour area settings is shown. Wrong positive structures (immune cells contours red; highlighted by arrows) area detected as possible organoid structures. An adaption of the contour area (parameter adaption: remove contours below 20 μm^2^) revealed an optimal detection of the organoid contours (orange area and red contour line). The bars represent 100 μm. Middle row: based on the contour area of organoid seeds (x-axis) and their corresponding intensity (y-axis) potential seeds candidates (inner area) can be determined for subsequent organoid detection. The upper right gate includes potential seeds candidates highlighted in red by back gating. These structures are further processed for organoid detection.

To improve the identification and quantification of organoid structures in lymphocyte co-cultures, we developed a StrataQuest-supported Organoid App. This software allows the processing of different image configurations and formats, ranging from time-lapse to end-point analysis of organoid development in co-cultures. In this context, an appropriate image sample identification code (ID) is mandatory for automated StrataQuest analysis, regardless of the image source. This ID must contain information about the plate number, well number, stimulation, and time point. Imported images obtained from alternative systems, such as Incucyte®, exhibit the following characteristics: 8-bit depth, resolution of 1,536 × 1,152 pixels, and a file size of approximately 4.5 MB. Consequently, a microscope field of view (FOV)-correction to 2,560×2,000 pixel must be performed by the StrataQuest software before starting the analysis. A simplified workflow of the organoid detection is summarized below and visualized in Figure 3.

#### 3.2.1 Generation of a virtual channel

The input image is converted into a virtual grayscale image (Figure 3A; Step #1). This step is mandatory for an optimal signal (high grayscale-values) to noise (low grayscale-values) separation. The contours of organoids with high contrast intensities become prominent and represent the key element for subsequent organoid detection, as well as for separating the inner areas from the surrounding environment.

#### 3.2.2 Background correction

A median filter is employed to create a suitable background model that is then subtracted from the grayscale image (Figure 3A; Step #2). This helps to reduce or eliminate non-uniform and high background signals, such as those arising from immune cells and other non-organoid structures.

#### 3.2.3 Grayscale image enhancement

A membrane detection algorithm is used to identify the contour areas of putative organoid-like structures. The software automatically enhances the intensity and contrast of the organoid borders (Figure 3A; Step #3) and processes them for subsequent verification and detection. After converting the original image to grayscale (Figure 3A, Step #2), the contours of the organoids exhibit a high level of intensity. As a result of this image processing, the membrane shows a higher grayscale intensity compared to the rest of the structures, including background and immune cells. This allows the algorithm to search for changes in intensity (higher or lower) within a defined set of growth steps. Finally, the algorithm detects shifts in intensity from a lower to a higher value and then back to a lower value and can define the shapes of maximum intensities that represent the border structure of the organoid. The output of this process is a black and white masked image in which the white areas represent the membrane-like structures identified by the membrane detection algorithm. The grayscale image is then supplemented with the identified membrane in order to highlight the contours of the organoids. This enhancement produces a refined grayscale image, utilized as the input for training the classifier.

#### 3.2.4 Classifier training by machine learning

Machine learning refers to the classification engine operated by the Organoid App, which can use representative images for each complexity category defined by the user. The machine learning process focuses on the dissection of real “organoid contour” and non-organoid structures, such as immune cells, which we call “tissue” in this publication. The enhanced grayscale image was used as the input image to facilitate the detection of the organoid contour (Figure 3A, Step #4; Supplementary Figure S2). After providing possible organoid boundaries and structures, the classifier is trained to identify all structures that are “real organoids”. In parallel, the classifier recognizes a so-called “tissue” that consists of immune cells and/or background. Various features such as intensity, morphology, Haralick texture, and environmental context, including Gaussian and median filters, are used to train the classifier by marking structures of interest and contaminating background signals (Supplementary Figure S2). The results are displayed as coded map images, where each organoid structure is marked with a color corresponding to its assigned boundary (Figure 3A, Step #5). Real-time detection and visualization of organoids during classifier training enables quality control of the classifier. The machine learning program supported by the Organoid App can be repeated until optimal recognition of organoids is achieved in the selected training images.

#### 3.2.5 Organoid contour and background detection

The contour mask (generated by the classifier) is highlighted in blue (Figure 3A; Step #5). Each disjunct object which generates a contour/organoid seed is assigned a unique image ID. The term “seeds” refers to an initial value that is used to initialize a random number generator algorithm, which then generates a sequence of random numbers based on that seed. These contour/organoid candidates are evaluated by morphological and intensity-based measurements (Figure 3A; Step #5). The background detection (highlighted in orange) allows the subtraction of structures that are not associated with organoids. This mask (including immune cells and debris), which is generated by the classifier, assigns a unique processing ID to each disjunct object, generating candidates for organoid seeds (Figure 3A; Step #6). The background mask (generated by the classifier) is labeled in red and separated from the actual organoid inner areas based on size criteria (Figure 3A; Step #7).

#### 3.2.6 Organoid body detection (seeds; inner area)

To visualize organoid bodies, seeds can be used to generate random gray intensities or patterns for labeling and distinguishing different organoids or regions of interest within an image. These candidates are evaluated using morphological and intensity-based measurements. To implement this process, the background gate (Figure 3A; Step #7) is used for subtracting the background for subsequent analyses. Based on the area (indicated gate) a unique ID is assigned to each disjunct object. Contours of the organoids are also used to validate the seeds (green contour; Figure 3A; Step #8). The candidates (Figure 3A; Step #9) are evaluated by morphological and intensity-based measurements, by two subsequent intervals. First, raw organoid body selection is performed by selecting the small tissue objects, which might represent potential organoids inner areas. Second, a more accurate organoid seed selection is performed by evaluating the intensities around the seeds from the previous step. A valid seed is surrounded by a calculated organoid contour (compare diagram: Seed candidate contour area (Figure 3B; Step #10, upper right quadrant)). The selected seeds are processed further using morphological operations (compactness) to better represent the inner area of organoids (Figure 3B; Step #11).

#### 3.2.7 Organoid detection and parameter adaption

The identified seeds (compare diagram: Organoid candidate area (μm^2^) vs. compactness; (Figure 3B; Step #11)) are combined with small contours, corresponding to small organoids where the inner area identification is very difficult or impossible. Organoid structures can now be detected based on two criteria: body; highlighted in green (Figure 3C; Step #12) and contour; highlighted in yellow (Figure 3C; Step #12). The resulting organoids are highlighted by the Organoid App in randomized colors (Figure 3C; Step #13). If the detection of organoids within the group of selected images is insufficient regarding FN or FP organoid structures, the classifier can be retrained again to achieve optimal automated detection of organoid candidates.

#### 3.2.8 Growing mask adaption and deletion of background signals

The raw organoid seed detection is combined with the contour mask, generated by the classifier, to create a growing mask (Figure 3D). Reconstruction of the organoids is performed by a growing algorithm that determines the final seeds on the growing mask. The growing step limits are defined by the parameter “Organoid – Grow Radius adaptation” (Figure 3D). For a precise adaption of organoid detection, it is also possible to adjust distinct growing masks accordingly. Additionally, an appropriate contour-size adaption enables the exclusion of background signals (Figure 3D). The identified organoids can be automatically categorized according to distinct areas.

## 4 Results

### 4.1 Quality control

The Organoid App was developed based on images of organoid co-cultures with immune cells (Figure 2A; level #1). Therefore, the default settings of the machine learning engine can be used to start the organoid detection procedure. Based on the heterogeneous quality of images and the varying density of organoids within cell culture plates, a project-specific adaption of the machine learning classifier is recommended to increase the quality of analysis.

**Figure 4:**
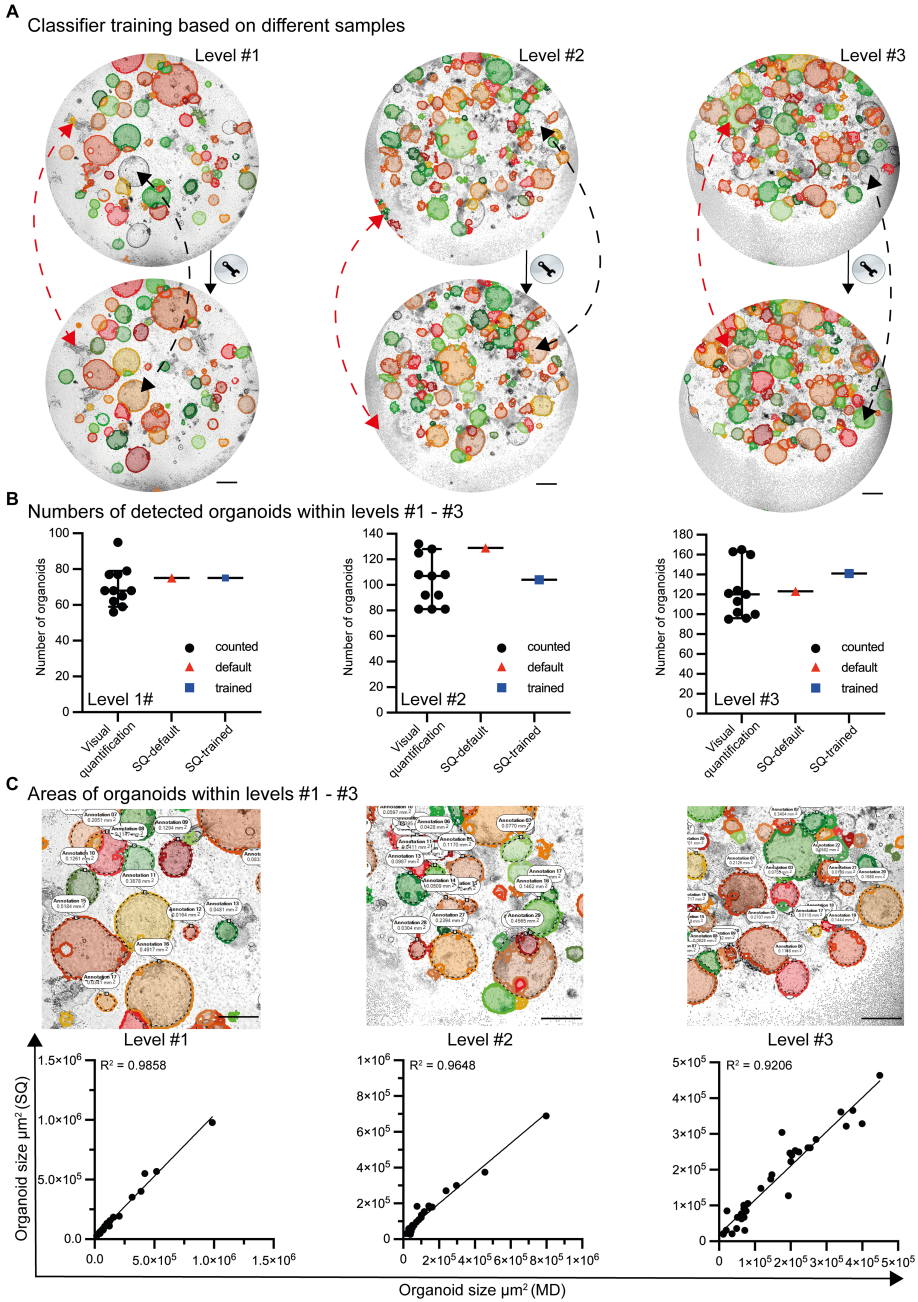
Classifier training and quality control by the StrataQuest-supported Organoid App. **(A)** Images with different levels of complexity (level #1 to #3) were analyzed by the default settings of the Organoid App (upper row) and after machine learning-based (lower row) classifier training. Representative modifications are highlighted by red and black dotted lines. The red lines indicate corrections of formerly false positive structures. The black lines indicate corrections of formerly false negative structures. The bars represent 500 μm. **(B)** The number of organoids is presented according to the indicated method of analysis. Visual quantification: the median and 95% confidence interval is shown. Each symbol represents an individual counting result (compare Figure 2). StrataQuest [(SQ)-default and SQ-trained: Each dot represents an analysis]. **(C)** The quality of area measurement is shown. Upper row: Areas detected by the StrataQuest based Organoid App (filled structures) and manually delineated boundaries (annotations and dotted black lines) of organoids. Representative images of different complexities (levels #1 to #3) are depicted. The bars represent 500 μm. Lower row: Linear regression plots, comparing the areas of organoids in μm^2^. The x-axis indicates the areas of manual delineation (MD). The *y*-axis indicates the areas of automated analysis performed by StrataQuest (SQ) using the Organoid App. Images with different complexities (level #1 to #3) were analyzed. A linear regression model was used to calculate the value of variation between the data. The R^2^ values are depicted.

Even though the default settings for organoid detection allow the identification of most organoids, some are not recognized (false-negative). In addition, organoid-like structures may be incorrectly identified (false-positive) as organoids (Figure 4A; top row). After appropriate classifier training and machine learning, the ability to recognize organoids in co-cultures is further improved (Figure 4A; lower row). Based on the high-throughput analysis of growing organoids from day 0 to day 6, the organoids exhibit varying densities and structures. Due to the significant variability and artifacts present in culture plates, the Organoid App has only very minor limitations, with high values of precision and recall (level #1 precision = 0.92/recall = 0.95, level #2 precision = 0.91/recall = 0.91, level #3 precision = 0.95/recall = 0.93; data not shown). This indicates that the Organoid App correctly identifies the majority of organoids. The identification and separation of overlapping organoids and technical artifacts remains indeed a real challenge, but has been largely solved with this application (Figures 4A,B).

The number of organoids detected by both the standard (StrataQuest (SQ)-default) and trained (SQ-trained) algorithm lies within the 95% confidence interval of the manually counted organoid numbers (Figure 4B). This result can be explained by the fact that after classifier training the number of false positives structures decreased (compare Figure 4A red arrows), whereas the detection rate of previously undetected organoids increased (compare Figure 4A black arrows).

In addition to the validation of the organoid detection count, the accuracy of the organoid area determination was verified. Randomly selected organoids were manually delineated and measured. These data were compared with the areas calculated by the Organoid App recognition algorithms. The corresponding linear regression graphs revealed a highly significant correlation between the Organoid App-based and manual-area measurements (Figure 4C). Our organoid application achieves a high degree of accuracy in organoid detection, closely paralleling the accuracy of the human eye (Figure 4C; Supplementary Figure S3B).

### 4.2 Characterization of organoid development within co-cultures in the presence of exogeneous compounds

A pilot study was conducted to evaluate the ability of the Organoid App to identify an aberration in organoid development under different growth conditions. In this context, we focused on two central aspects. First, we tested whether organoid growth can be monitored over time. Second, we addressed whether the simultaneous presence of a growth factor and immune cells can affect organoid differentiation in terms of size and compactness. To this end, we have included the epidermal growth factor (EGF) in our study, as EGF has already been used in a number of studies on organoid differentiation (27, 33–36).

**Figure 5:**
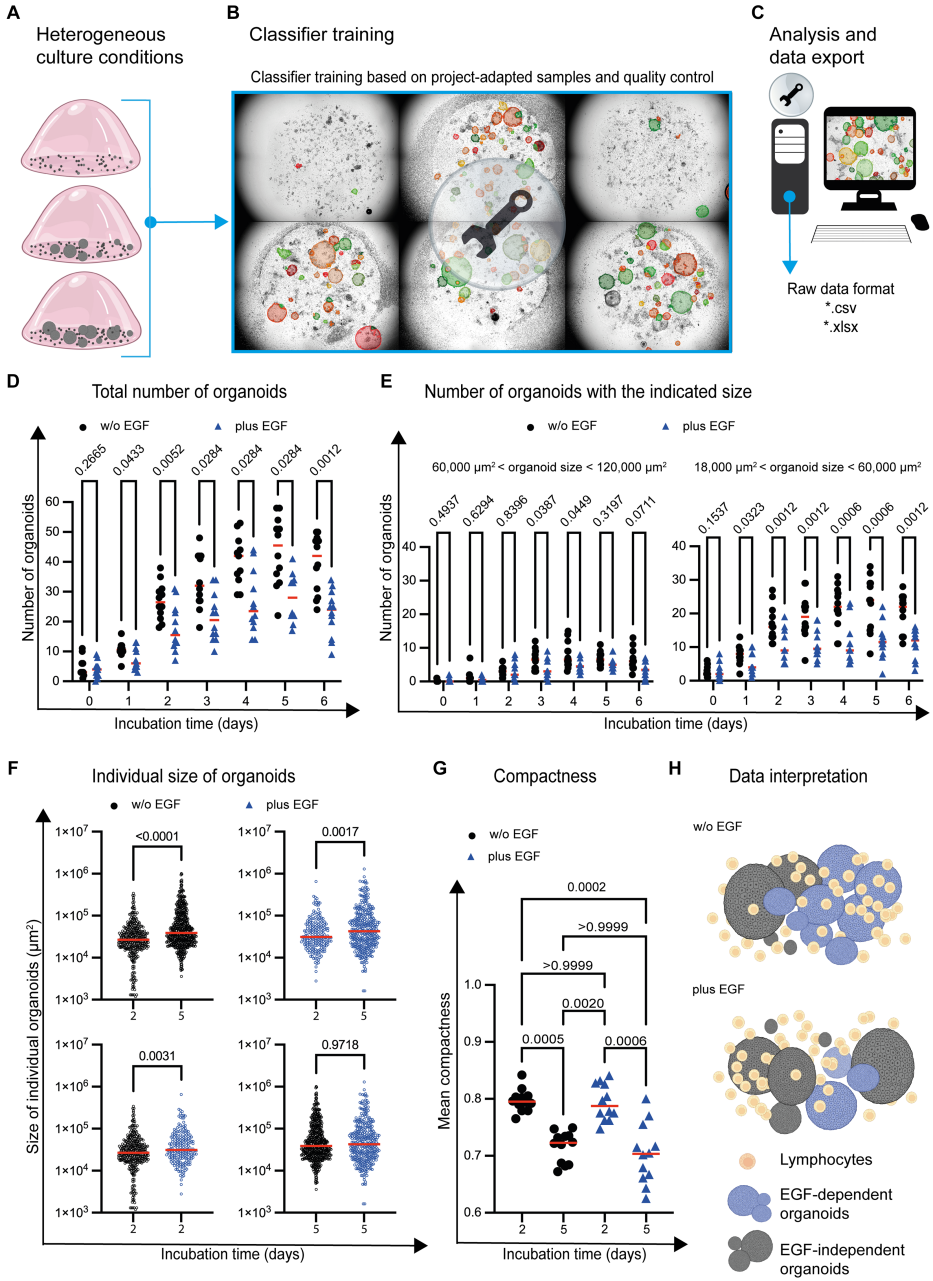
Organoid development in the presence of immune cells and EGF. **(A)** Graphical examples of different organoid growth conditions are depicted. **(B)** Based on the given complexity, the classifier is trained by using images (*n* = 6) representing the range of the given cell culture complexity. **(C)** The analysis of the entire project (*n* = 170 images) is based on the project-specific classification and detection settings. **(D)** The total number of organoids and **(E)** organoids within a distinct size range are depicted. Each symbol represents the total number of organoids within one well. Quadruplicates from *n* = 3 different donors are shown. The red horizontal line highlights the median. Multiple *t*-test (Multiple Mann–Whitney tests; unpaired; nonparametric) have been used to compare organoid cultures in the absence (w/o EGF; black circle) and presence (plus EGF; blue filled triangle) of EGF. The *p* values are indicated. **(F)** The individual size of organoids at day 2 and day 5 is shown. Each dot represents the size of one single organoid within the culture wells. The horizontal line highlights the median. Quadruplicates from *n* = 3 donors are shown. Multiple *t*-test (Multiple Mann–Whitney tests; unpaired; nonparametric) have been used to compare organoid co-cultures in the absence (w/o EGF; blue circle) and presence (plus EGF; blue circle) of EGF. The *p* values are indicated. **(G)** The mean of organoid candidates’ compactness at day 2 and day 5 is shown. Each dot represents the area of a single organoid. The horizontal line represents the median. Quadruplicates from *n* = 3 donors have been implemented in the data analysis. One way ANOVA tests (Kuruskal-Wallis test, Dune’s multiple comparison) have been used comparing organoid cultures in the absence (w/o EGF; black circle) and presence (plus EGF; blue filled triangle) of EGF. The *p* values are indicated. **(H)** The diagram illustrates a potential interpretation of the data: The development of organoids with an intermediate size (highlighted in blue) between 18,000 μm^2^ and 60,000 μm^2^ is reduced in the presence of EGF. The growth of the blue organoids is EGF-dependent (EGF-dependent organoids). Gray organoids can grow in the absence of EGF (EGF-independent organoids).

Images were acquired over a period of 6 days with the Incucyte® SX5 Live-Cell Analysis System and imported into StrataQuest. An initial screening of the data revealed that the number and size of the organoids changed significantly over the course of a 6-day culture period. Even the density of immune cells in the *in vitro* system exhibited substantial fluctuations due to cell proliferation, as illustrated in Figure 5A. To ensure accurate detection of organoids by the Organoid App, we trained the classifier on a set of six representative images. These selected images were imported and merged into a single dataset. Based on this dataset, it was feasible to achieve a comprehensive training covering the full range of cell culture heterogeneity, including organoid density, overlapping structures and imaging quality (Figure 5B). After appropriate classifier training, image analysis was conducted.

StrataQuest allows the export of meta data in different file formats such as *.pdf, *.xls and *.xlsx for subsequent graphical display and statistical analysis (Figure 5C). This enables the import and further processing by other software tools. Consequently, adequate statistical analyses and data visualization can be performed. Our analysis showed an increase in the absolute number of organoids over time, both in untreated and EGF-treated culture conditions (Figure 5D). However, the total number of organoids was reduced in the presence of EGF (Figure 5D).

To determine whether the development of organoids with a distinct size was affected by EGF, organoids of different sizes were analyzed. This characterization revealed that the number of organoids (day 1–day 6) with a size between 18,000 μm^2^ and 60,000 μm^2^ is impaired in the presence of EGF (Figure 5E). The number of organoids with a size between 60,000 μm^2^ and 120,000 μm^2^ was only partially (day 3 and day 4) affected by EGF (Figure 5E). In contrast, EGF does not influence the development of organoids below 18,000 μm^2^ or above 120,000 μm^2^ in general (Supplementary Figure S4).

The Organoid App also enables the determination of individual organoid sizes within the co-culture system. Comparing day 2 with day 5 of organoid co-culture, the average size of organoids increased, independent of EGF (Figure 5F; upper dot plots). We also analyzed the influence of EGF on organoids growth on day 2 and 5 and found that while EGF had no effect on the size distribution of organoids on day 5 (Figure 5F: lower right dot plot), on day 2 EGF led to a very slight increase in average organoid size (Figure 5F: lower left dot plot).

In addition to the quantification of organoid number and size, the compactness of individual organoids can be determined. Compactness can be assessed using various quantitative metrics, such as the ratio of the organoid’s volume to its surface area or the degree of sphericity. These measurements provide information about the overall structural integrity and density of organoids. A high compactness value indicates a well-developed cohesive circular organoid structure, while a lower value may indicate a diffuse arrangement of cells within an amorph organoid. We found increased compactness at day 2 compared to day 5 (Figure 5G), indicating that the organoids become less compact during culture.

Based on these first proof-of-principle experiments, it can be concluded that a sound quantification of organoid number, size and compactness in co-cultures with immune cells is possible by using the Organoid App in high-throughput analysis. Our study also confirms that the Organoid App effectively detects subtle differences in the chosen culture conditions. We found that EGF has the potential to affect the development of distinct organoid subsets (Figure 5H).

**Figure 6:**
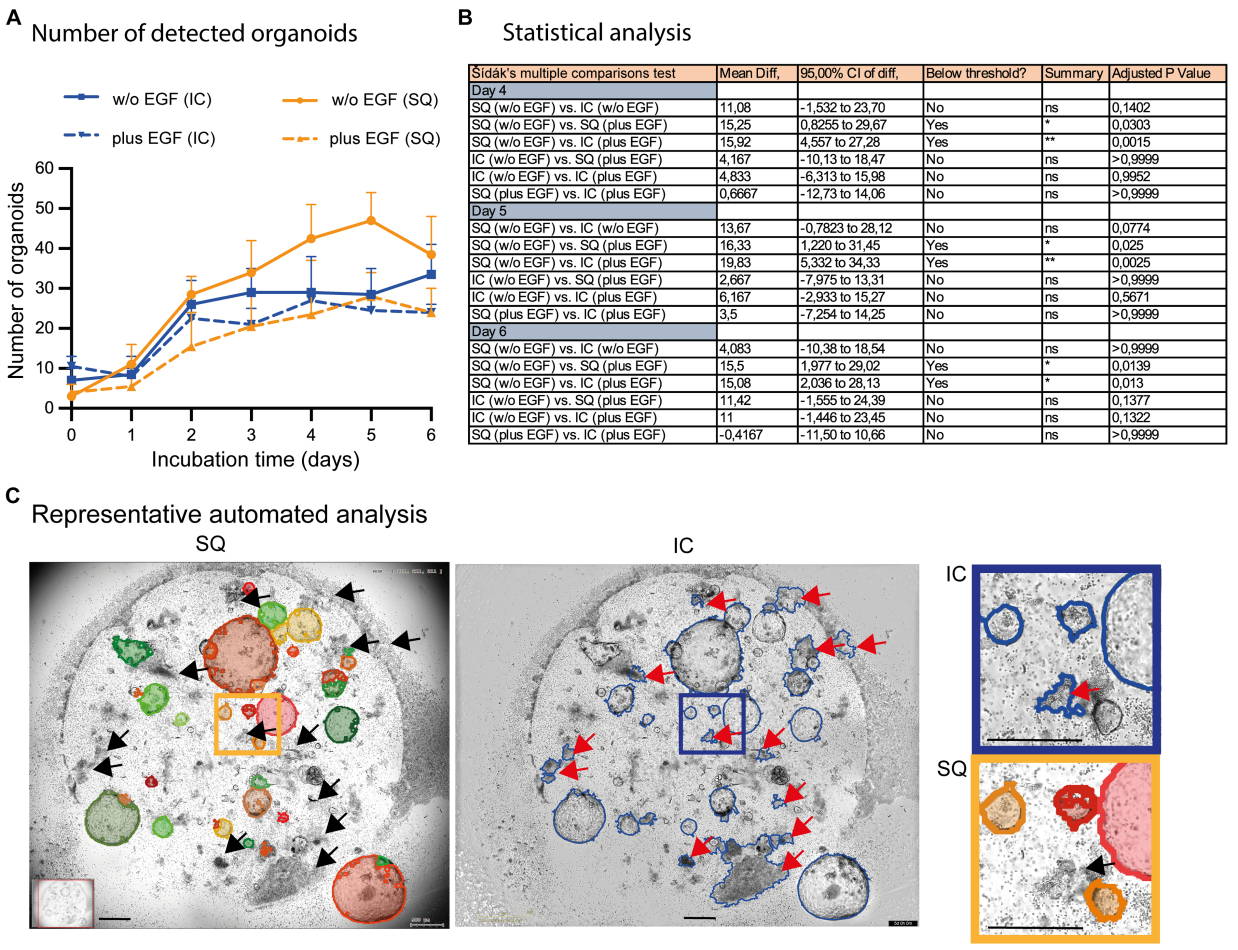
Comparison of automated organoid detection applications. **(A)** The total number of organoids determined by the StrataQuest Organoid App (SQ; orange lines; plus, EGF solid, w/o EGF dotted) and Incucyte^®^ (blue lines; plus EGF solid, w/o EGF dotted) is shown. The median from quadruplicates of *n* = 3 different donors plus the 95% confidence interval is depicted. **(B)** The Šídák’s multiple comparisons test highlights the statistical data of selected samples. **(C)** Representative analysis (day 5 w/o EGF) are shown. Left: StrataQuest-supported Organoid App (black arrows highlight correct exclusion of organoid- mimicking contours). Middle: Incucyte^®^ analysis (red arrows highlight wrong detection of organoid-mimicking contours). Right: Inserts with higher amplification are depicted. The bars represent 500 μm.

We have also incorporated the Incucyte® software for subsequent quantification of organoids in co-cultures, despite its obvious limitations in organoid detection (compare Figure 2B; right column). These data revealed that the Incucyte® software was not able to detect the differences in organoid growth between untreated and EGF-treated co-cultures, which could only be identified by the Organoid App (Figures 6A,B). Due to the limitations of the Incucyte® software (Figure 6C), these data cannot be used to further interpret the effect of EGF on organoid growth. The Organoid App, powered by StrataQuest and capable of detecting and quantifying of organoids, showed that EGF can dampen the growth of organoids in co-culture systems (Figures 6A,B). These data are consistent with the data shown in Figure 5D. Further analysis and comparison of organoid growth with OrganoSeg was not performed due to the limitations of this algorithm in organoid detection.

## 5 Discussion

The debate on whether or not engineered cell-based *in vitro* models, such as organoids, can faithfully reproduce the structures and functions of the original organ *in vivo* is ongoing (37, 38). Although organoid systems are limited in their ability to mimic the properties of complex *in vivo* tissues relevant to physiological or pathological processes in tissues, organoid cultures are still useful tools for studying fundamental mechanistic questions (39). Healthy and pathologically altered tissues, however, are composed of different cell types. Therefore, adding tissue-specific stromal or immune cells to organoid cultures may improve the physiological context of organoid development. Developing multicellular organoid models that better represent *in vivo* micro-environments is still challenging, but progress is already evident (39–42).

In addition to the complexity of the multicellular organoid model system itself, the strength of the analytic data is influenced by the image quality and structural diversity of the acquired objects. Even when image quality is excellent, the subsequent processing is still limited by other factors such as contour mimicry, fusion, and superposition of structures. These artifacts may result in the incorrect identification of organoid structures causing false negative or false positive data and therefore low values of recall and precision. Consequently, it is crucial to display organoid boundaries with maximum contrast to achieve clear visual delineation, which can be accomplished with our newly developed Organoid App.

Although machine learning classifiers are highly sophisticated, technical limits remain. We do not believe that any currently available algorithm will accurately detect 100% of the target structures since optical structure recognition involves the analysis and interpretation of visual data. While significant progress has been made in the fields of computer vision and image processing, achieving “100% accuracy” in optical structure detection remains a theoretical ideal rather than a practical expectation.

Several challenges hinder the achievement of perfect accuracy in optical structure detection. Artifacts and variability within optical data, caused by imaging conditions like illumination or sensor limitations, can lead to significant detection errors. Complex and overlapping optical patterns and structures make an accurate detection difficult, potentially resulting in incorrect outcomes. Algorithm limitations also pose challenges, as diverse algorithms have individual strengths and weaknesses and may not cover all scenarios effectively. Additionally, human subjectivity in defining and recognizing optical structures introduces variability due to differences in expert criteria for setting detection thresholds.

Despite this technical hurdle, our data demonstrates that the Organoid App can accurately detect organoids in co-cultures. This was achieved by separating the organoid structures from the other contaminating structures such as immune cells and imaging artifacts. Even though the Organoid App occasionally fails to correctly recognize a minority of overlapping structures, most of them are detected correctly. In contrast to other software solutions such as Incucyte® or OrganoSeg, we were able to precisely detect organoids in co-cultures with immune cells for the first time. Therefore, we utilized the Organoid App to examine organoid growth under varying conditions. In this context, we questioned whether the Organoid App is able to highlight differences regarding the growth of organoids under the given co-culture conditions.

The term “growth of organoids” can be used to either address the numerical expansion of organoid structures, or the increase in size of a single organoid. Organoids are usually derived from progenitor cells which have the potential to differentiate into complex and self-organized structures (7, 43, 44). Exactly these early multicellular organoid precursors may be different in terms of their potential for organoid formation and subsequent growth. Accordingly, it can be assumed that the growth of individual organoids is determined by their cellular composition and the ability of the respective precursor cells to form organoids (2). Thus, the number of detected organoids can reflect the potential of organoid precursors to replicate and to survive under the given conditions. The increase in organoid size indicates the potential of organoid associated cells to replicate. In order to make a statement about organoid growth, both parameters should be taken into account. It is quite possible that the number of organoids can increase without an expansion in size. To confirm or exclude heterogeneity in organoid growth, it is also necessary to consider the parameter of individual organoid sizes within the co-culture. Various organoid-associated parameters, such as quantity, average size, and individual size, and compactness might therefore offer a promising approach for a more detailed characterization of organoid development within culture conditions.

Due to these facts, we evaluated time-lapse images of organoid co-cultures with immune cells in order to determine the effect of the growth factor EGF on organoid development. Based on the data we have generated with the Organoid App, we can conclude that organoids can be formed and detected under the given culture conditions. However, EGF is capable of suppressing organoid growth within the selected culture conditions. While the effect of EGF on ECO differentiation and growth has not been analyzed yet, there are a variety of reports on the use of EGF for organoid differentiation in the absence of differentiated immune cells (27, 33–36). Our results show that the combination of immune cells and EGF results in a reduced expansion of organoids within the co-culture. In this context, it is noticeable that the total number of organoids with a distinct size is reduced. This suggests that certain organoids are still able to grow effectively in the presence of EGF. The increase in organoid size could be due to increased cell-proliferation within the organoid bodies and/or accumulation of extracellular matrix components within the organoid structures. Thus, it can be assumed that individual organoids react differently to EGF. This effect might be explained by the fact that the composition of organoids regarding stem cells and other cell subsets is not homogeneous. While we could detect an increase in organoid size between day 2 and 5, regardless of the presence of EGF, the size of individual organoids was increased slightly in the presence of EGF on day 2 but not on day 5. In addition, we evaluated the compactness of the organoids in culture and found a reduction of compactness on day 5 compared to day 2, independent of the presence of EGF. This is an indication of a possible transformation to less “circular” organoids.

The initial pilot experiment studying the effects of EGF on organoid development in co-cultures with immune cells yielded these primary results. EGF shows a detrimental impact on individual organoids over the culturing period, leading to a decrease in the number of organoids of certain sizes. However, despite this reduction, certain organoids within the co-culture exhibit an increase in size when exposed to EGF. Hence, EGF appears to impact the early-stage organoid development in co-culture with CD8^+^ effector T cells, leading to a reduced proportion of organoids capable of growth under specific immune cell culture conditions. It is known that conventional human T lymphocytes do not express the EGFR (45). However, distinct differentiated T-cell subsets such as regulatory T cells, can express the EGFR and benefit from other EGFR ligands such as amphiregulin (46, 47). The expression of EGFR by T cells that are in close proximity to organoids, as well as their potential role in regulating EGF-dependent organoid formation, remains unknown and warrants further investigation.

In summary, our presented high-throughput imaging solution offers great potential for further analysis and detection of organoids within co-culture systems. The Organoid App can be helpful in the development of innovative diagnostic and therapeutic strategies by enabling the study of organoids in the presence of human immune cells and exogenous substances such as drugs or cytokines.

## Data availability statement

The original contributions presented in the study are included in the article/Supplementary material, further inquiries can be directed to the corresponding author.

## Ethics statement

The studies involving human samples were approved by Ethical committee (Regensburg University). Organoids: Regensburg University, reference number reference number 16-101-5-101. CD8 T cells: Regensburg University, reference number 13-0240-101 and 19-1414-101. The studies were conducted in accordance with the local legislation and institutional requirements. The participants provided their written informed consent to participate in this study.

## Author contributions

PS: Methodology, Software, Visualization, Writing – review & editing. BN: Data curation, Formal analysis, Investigation, Methodology, Software, Validation, Writing – review & editing. SH: Data curation, Formal analysis, Writing – review & editing. MW: Methodology, Software, Writing – review & editing. MF: Methodology, Software, Writing – review & editing. HJ: Funding acquisition, Writing – review & editing. EE: Validation, Writing – review & editing. BL: Methodology, Software, Writing – review & editing. SL: Methodology, Software, Writing – review & editing. UR: Conceptualization, Data curation, Formal analysis, Investigation, Methodology, Software, Supervision, Validation, Visualization, Writing – original draft, Writing – review & editing.

## Footnotes

^1^ https://github.com/JanesLab/OrganoSeg.git

^2^ https://github.com/JanesLab/OrganoSeg/blob/main/BortenMA_OrganoSeg_Readme_GitHub.pdf
