# Supplementary material for: Analysis of organoid and immune cell co-cultures by machine learning-empowered image cytometry
Source: Front Med (Lausanne). 2024 Jan 17;10:1274482. doi: 10.3389/fmed.2023.1274482 (PMC10827864; doi:10.3389/fmed.2023.1274482)
Supplement: Supplementary file 1 [file Image_1.pdf]

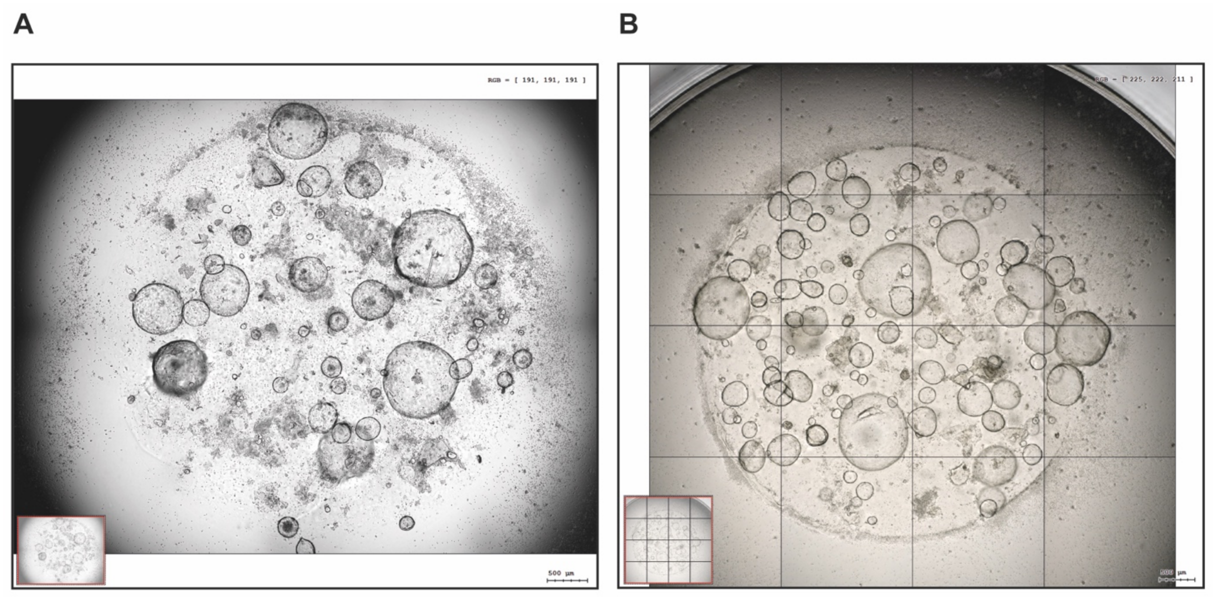

Supplementary Figure 1: Visualization of organoids embedded in Matrigel® domes using different devices.

Representative images acquired by different imaging systems are shown. (**A**) Incucyte®: brightfield organoid scan mode, object driven focus, z-depth < 2.9 mm (29). (**B**) TissueFAXSiPLUS: Merged z-stacks (*n = 4*, range 310 µm). The black quadrants highlight the boarders of stitched single images. The bars represent 500 µm.

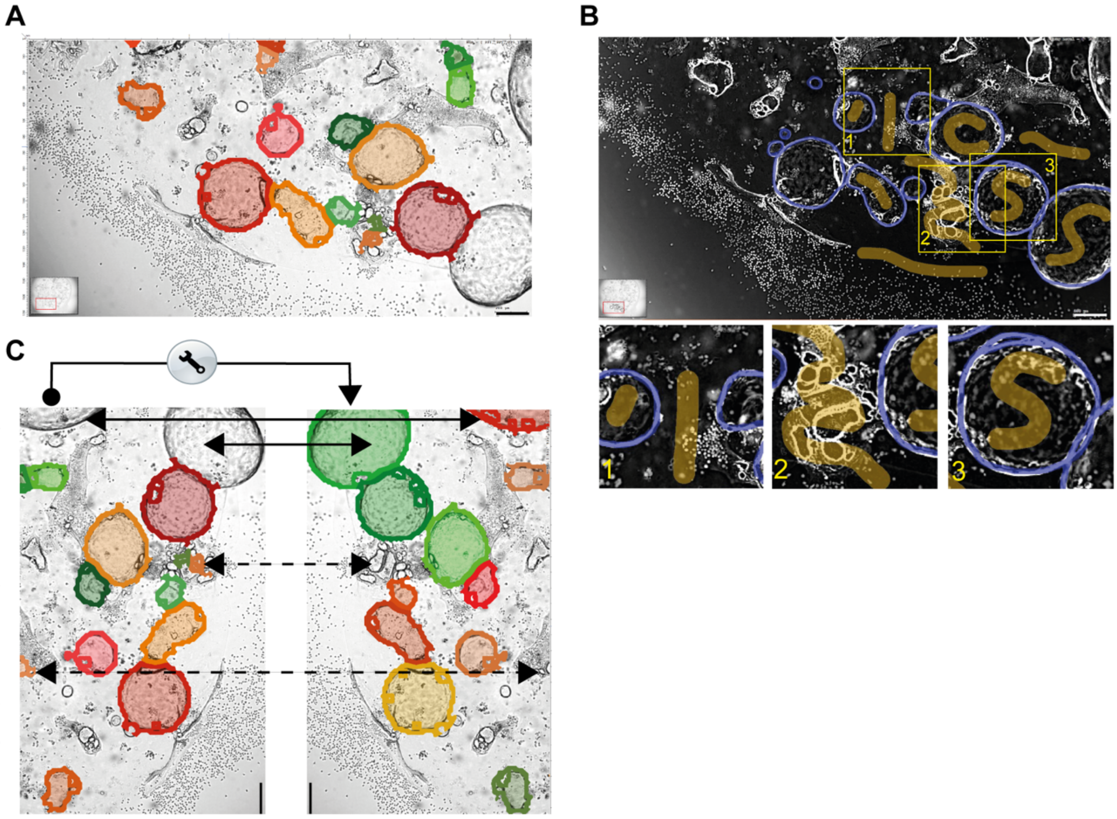

Supplementary Figure 2: Classifier training and machine learning.

(**A**) The RGB image shows organoids (colored filled structures) that have been detected using the default detection settings (organoid growth radius 90,00 µm and organoid seed growth radius 290,00 µm). Structures above 5,000 µm^2 have been included for subsequent classifier training (bar = 200 µm). (**B**) An Organoid App generated inverted greyscale image is shown. The blue and yellow filled lines represent the regions marked with the cursor, which are used for the subsequent training of the classifier (organoid contours: filled blue lines; background: filled yellow lines). The inserts 1-3 show the representative training areas in more detail (bar = 200 µm). (**C**) Mirrored images are mapped for easier tracking of changes to RGB images. Left side: Before classifier training. Right side: After classifier training. Solid arrows highlight false negative organoid structures (left side) that are recognized by the algorithms after the classifier training (right side). Dotted arrows highlight false positive organoid structures (left side) that are removed by the Organoid App after appropriate classifier training (right side). The bars represent 200 µm.

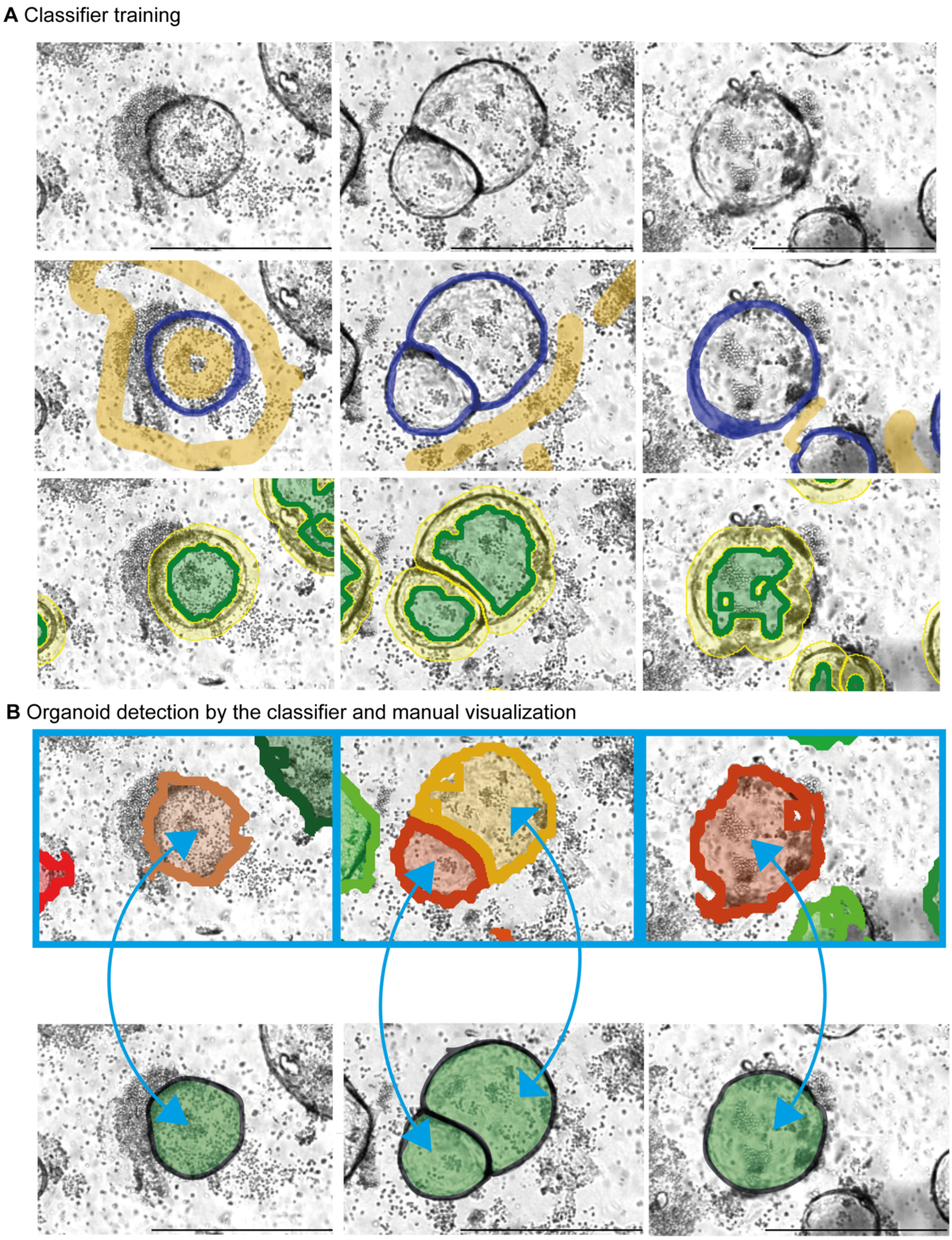

Supplementary Figure 3: Challenging tasks and correct detection of organoids by the Organoid App.

ECOs and immune cells, embedded in Matrigel®, were co-cultured for a period of 6 days. Image acquisition was performed in the z-depth acquisition mode. The Organoid App has been applied for the detection of organoids by StrataQuest. (**A**) An overview of the classifier training is depicted. Upper row: original images. Middle row: classifier training. To train the classifier, the

organoid boundaries (blue line) and the background (orange line) were marked. Lower row: the results of potential organoid candidates are depicted. The Organoid App-defined organoid structures are depicted. Yellow lines indicate the boundaries of the organoid, and the filled green area represents the surface of the organoid. The bars represent 500 µm. (**B**) Organoid detection by the classifier (upper row) and manual (lower row) visualization (compare Figure 1F). Upper row: individual organoids are highlighted by the Organoid App. The transparent area shows the surface of individual organoids. The filled lines mark the boundaries of each organoid. Lower row: the aimed correct detection of organoids is highlighted in green. The bars represent 500 µm. Blue arrows indicate computed and manually marked organoids.

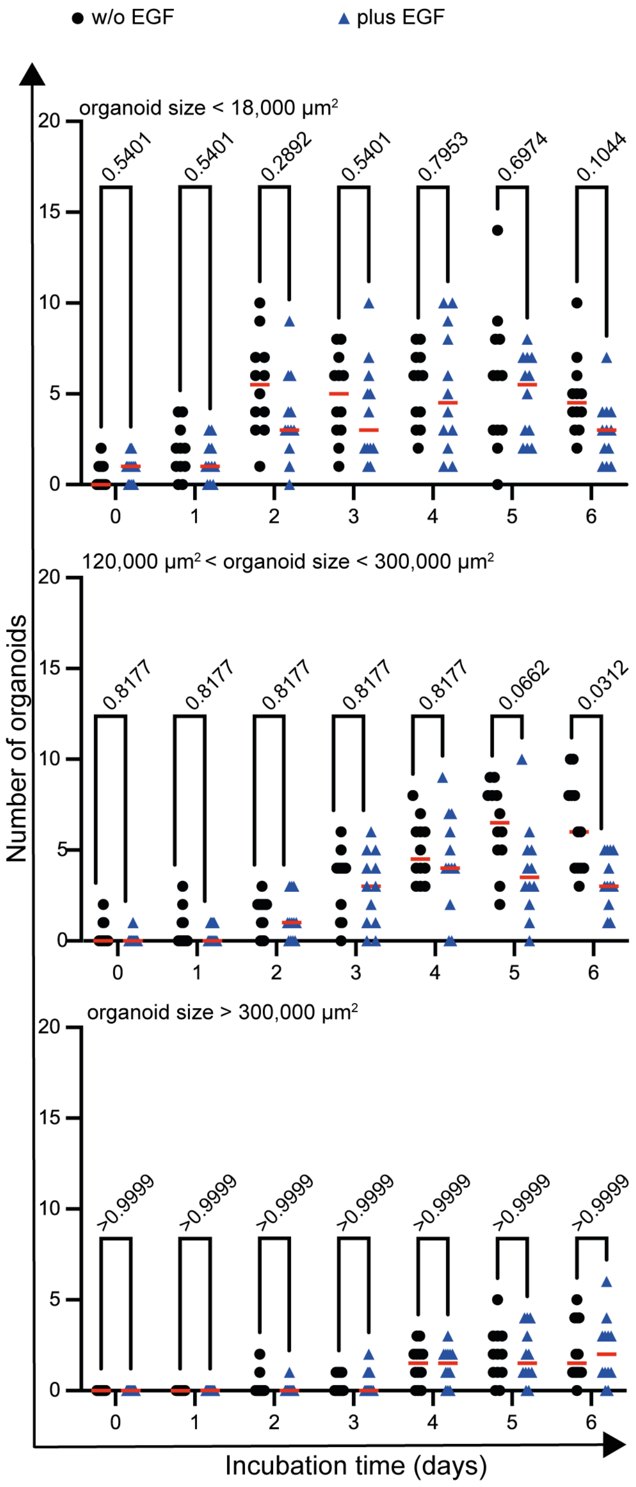

Supplementary Figure 4: Organoid development in the presence of immune cells and EGF.

The total number of organoids with distinct sizes are depicted. Each symbol represents the total number of organoids within one well. Quadruplicates from *n = 3* different donors are shown. The red horizontal line highlights the median. Multiple t-tests (Multiple Mann-Whitney tests; unpaired; nonparametric) have been used comparing organoid cultures in the absence (w/o

EGF; black circle) and presence (plus EGF; blue filled triangle) of EGF. The p values are indicated.
